# *tert*-Butyl 6-bromo-1,4-dimethyl-9*H*-carbazole-9-carboxyl­ate

**DOI:** 10.1107/S1600536810026528

**Published:** 2010-07-10

**Authors:** Jean-François Lohier, Anna Caruso, Jana Sopková-de Oliveira Santos, Jean-Charles Lancelot, Sylvain Rault

**Affiliations:** a Laboratoire de Chimie Moléculaire et Thio-organique, UMR CNRS 6507, FR CNRS 3038 INC3M, ENSICAEN – Université de Caen, 14050 Caen, France; b Centre d’Études et de Recherche sur le Médicament de Normandie (CERMN), EA 3915, FR CNRS 3038 INC3M, Université de Caen, boulevard Becquerel, 14032 Caen, France

## Abstract

The title compound, C_19_H_20_BrNO_2_, consists of a carbazole skeleton with methyl groups at positions 1 and 4, a protecting group located at the N atom and a Br atom at position 6. The pyrrole ring is oriented at dihedral angles of 1.27 (7) and 4.86 (7)° with respect to the adjacent benzene rings. The dihedral angle between the benzene rings is 5.11 (7). The crystal structure is determined mainly by intra­molecular C—H⋯O and inter­molecular π–π inter­actions. π-stacking between adjacent molecules forms columns with a parallel arrangement of the carbazole ring systems. The presence of the *tert*-but­oxy­carbonyl group on the carbazole N atom and the intra­molecular hydrogen bond induce a particular conformation of the exocyclic N—C bond within the mol­ecule.

## Related literature

For the pharmaceutical properties of carbazole derivatives, see: Itoigawa *et al.* (2000 ▶); Laronze *et al.* (2005 ▶); Thevissen *et al.* (2009 ▶). For their electroactivity and luminescent properties, see: Grazulevicius *et al.* (2003 ▶) and for their their applications in the light-emitting field, see: Zhang *et al.* (2006 ▶). For the synthesis of carbazoles and ellipticine derivatives, see: Ergün *et al.* (1998 ▶); Knölker *et al.* (2002 ▶); Liu *et al.* (2007 ▶). For related structures, see: Caruso *et al.* (2007 ▶); Sopková-de Oliveira Santos *et al.* (2008 ▶). For bond-length data, see: Allen *et al.* (1987 ▶). The title compound constitutes a cheap and reactive inter­mediate for the preparation of new analogs of the anti­cancer agent 9-meth­oxy­ellipticine, see: Le Pecq *et al.* (1974 ▶). A lengthening of N—C bond lengths due to the presence of a protecting group has been observed in similar compounds, see: Back *et al.* (2001 ▶); Chakkaravarthi *et al.* (2009 ▶); Terpin *et al.* (1998 ▶) For *N*-sulfonyl carbazole derivatives with similar conformations, see: Chakkaravarthi *et al.* (2008 ▶). For non N-atom-substituted analogs, see: Viossat *et al.* (1988 ▶).
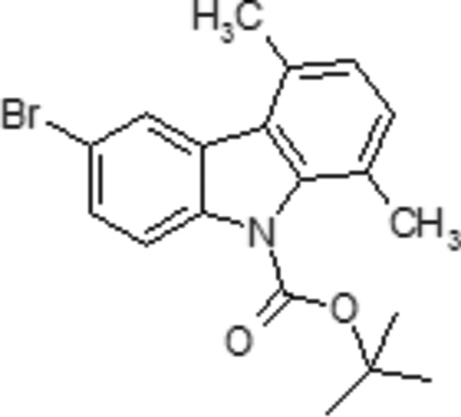


## Experimental

### 

#### Crystal data

C_19_H_20_BrNO_2_
*M* *_r_* = 374.27
Triclinic, 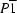

*a* = 7.521 (4) Å
*b* = 9.715 (5) Å
*c* = 11.930 (6) Å
α = 91.10 (4)°
β = 96.40 (4)°
γ = 90.96 (4)°
*V* = 865.9 (8) Å^3^
*Z* = 2
Mo *K*α radiation
μ = 2.38 mm^−1^
*T* = 291 K
0.46 × 0.37 × 0.34 mm

#### Data collection

Bruker–Nonius APEXII KappaCCD diffractometer
Absorption correction: numerical (*SAINT*; Bruker, 2007 ▶) *T*_min_ = 0.378, *T*_max_ = 0.429
37091 measured reflections
5718 independent reflections
4268 reflections with *I* > 2σ(*I*)
*R*_int_ = 0.025

#### Refinement

*R*[*F* ^2^ > 2σ(*F* ^2^)] = 0.034
*wR*(*F* ^2^) = 0.092
*S* = 1.02
5718 reflections
213 parameters
H-atom parameters constrained
Δρ_max_ = 0.63 e Å^−3^
Δρ_min_ = −0.52 e Å^−3^

### 

Data collection: *APEX2* (Bruker, 2007 ▶); cell refinement: *SAINT* (Bruker, 2007 ▶); data reduction: *SAINT*; program(s) used to solve structure: *SHELXS97* (Sheldrick, 2008 ▶); program(s) used to refine structure: *SHELXL97* (Sheldrick, 2008 ▶); molecular graphics: *Mercury* (Macrae *et al.*, 2008 ▶); software used to prepare material for publication: *SHELXL97*.

## Supplementary Material

Crystal structure: contains datablocks I, global. DOI: 10.1107/S1600536810026528/om2338sup1.cif

Structure factors: contains datablocks I. DOI: 10.1107/S1600536810026528/om2338Isup2.hkl

Additional supplementary materials:  crystallographic information; 3D view; checkCIF report

## Figures and Tables

**Table 1:** Hydrogen-bond geometry (Å, °)

*D*—H⋯*A*	*D*—H	H⋯*A*	*D*⋯*A*	*D*—H⋯*A*
C8—H8⋯O2	0.93	2.33	2.863 (3)	116

**Table 2:** π–π inter­actions (Å, °) *Cg*1, *Cg*2 and *Cg*3 are the centroids of the N9–C9*A*–C4*A*–C5*A*–C8*A*, C9*A*–C1–C2–C3–C4–C4*A* and C5*A*–C5–C6–C7–C8–C8*A* rings, respectively, ccd is the distance between ring centroids, sa is the mean slippage angle (angle subtended by the inter­centroid vector to the plane normal) and ipd is the mean inter­planar distance (distance from one plane to the neighbouring centroid). For details, see Janiak (2000 ▶).

Group 1/group 2	ccd	sa	ipd
*Cg*2/*Cg*3^i^	3.755 (2)	24	3.532 (1)
*Cg*3/*Cg*2^i^	3.755 (2)	20	3.433 (1)
*Cg*1/*Cg*1^i^	3.927 (2)	22	3.638 (1)
*Cg*2/*Cg*3^ii^	3.811 (2)	18	3.654 (1)
*Cg*3/*Cg*2^ii^	3.811 (2)	16	3.626 (1)
*Cg*1/*Cg*1^ii^	4.199 (2)	32	3.578 (1)

Symmetry codes: (i) −*x* + 1, −*y*, −*z* + 1; (ii) −*x*, −*y*, −*z* + 1.

**Fig. 1.**
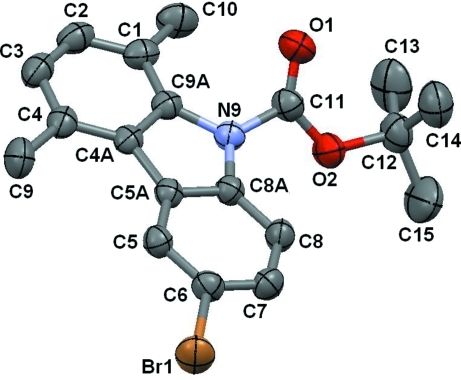
the title compound showing the atom-labelling scheme. Displacement ellipsoids are drawn at the 50% probability levels; For the sake of clarity H atoms have been omitted.

**Fig. 2.**
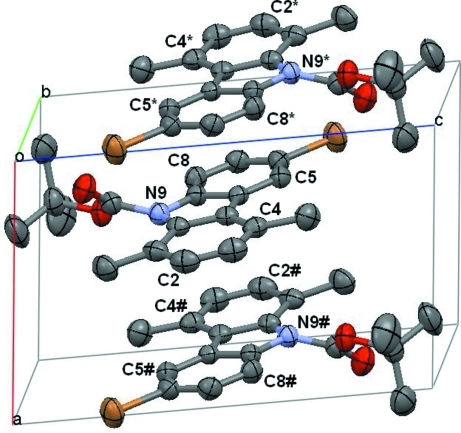
Part of the crystal packing showing the way in which a column along a axis is formed through π–π interactions. For the sake of clarity H atoms have been omitted. [Symmetry codes: (*)-x,-y, 1 - z; (#) 1 - x,-y, 1 - z.]

## supplementary crystallographic information

### Comment

Over the past few years, large interest has been observed in chemistry of carbazole derivatives since they can be widely used as organic materials due to their electroactivity and luminescent properties (Grazulevicius *et al.*, 2003) or their applications in the light-emitting field (Zhang *et al.*, 2006). This class of compounds also displays various pharmacological activities such as, among others, anticancer (Itoigawa *et al.*, 2000; Laronze *et al.*, 2005), antibacterial and antifungal activities (Thevissen *et al.*, 2009).

Many elegant methods for the synthesis of ellipticine and related carbazole alkaloids have been reported (Ergün *et al.*, 1998; Knölker *et al.*, 2002; Liu *et al.*, 2007). In our laboratory, the quest to discover new potential bioactive compounds possessing a carbazole core has attracted all our attention and recently, we have synthesized and characterized a series of carbazole derivatives (Caruso *et al.*, 2007; Sopková-de Oliveira Santos *et al.*, 2008). In this paper, we present the results of structural investigation of a new intermediate (Scheme 1): 6-bromo-9-*tert*-butoxycarbonyl-1,4-dimethyl-9*H*- carbazole (Fig. 1) which constitutes a very interesting, cheap and reactive intermediate for the preparation of new analogs of the anticancer agent 9-methoxyellipticine (Le Pecq *et al.*, 1974).

The carbazole ring system (C1—C9A/N9) is nearly planar and the maximum deviation from the least-squares planes does not exceed 0.0662 (14) Å. The pyrrole ring is oriented with respect to the adjacent benzene rings at dihedral angles of 1.27 (7) and 4.86 (7)°.

The N—C bond lengths, namely N9—C8A and N9—C9A [1.408 (2)Å and 1.417 (2) Å] deviate slightly from the normal mean value reported in the literature (Allen *et al.*, 1987). This indicates that the presence of protecting group at atom N9, probably through its electron-withdrawing character, causes the lengthening of N—C bond lengths which has been already observed with similar compounds (Back *et al.*, 2001; Terpin *et al.*, 1998; Chakkaravarthi *et al.*, 2009). Methyl substituent C9 is coplanar with the aromatic rings, methyl substituent C10 closed to N-protecting group displays slight deviation from the carbazole plane with torsion angle values C4A—C9A—C1—C10 of -172.72 (15). This is probably due to minimize the steric hinderance induced by the carbamate group. No particular increase in the widening angle, namely C9A—C1—C10, has been observed compared to non substituted nitrogen atom analogs (Viossat *et al.*, 1988). Weak intramolecular C—H···O interaction is present in the molecule. In fact, atom C8 acts, throught H8, as hydrogen-bond donor to O2, distance between H8 and O2 being 2.33 Å (Table 1). Thus, in order to optimize previous H-bond and minimize steric hinderance of N-protecting group, carbamate is forced to adopt a particular conformation, specially a very twisted torsion angle which have been also seen with *N*-sulfonyl carbazole derivatives displaying intramolecular H-bonds (Chakkaravarthi *et al.*, 2008). Thus, the torsion angle C1—C9A—N9—C11 is as high as 30.8 (2)°.

In the crystal packing, π–π interactions may be effective in the stabilization of the structure. Stacking interactions occur between aromatic rings leading to columns along *a* axis. The arrangement of carbazole ring systems within column is parallel but non equally spaced and molecules rotate of 180° alternatively. More precisely, π–π contacts are present with *Cg*2···*Cg*3 distance = 3.755 (2)Å [symmetry code: 1 - *x*,-y, 1 - *z*] and 3.811 (2)Å [symmetry code:-x,-y, 1 - *z*]. *Cg*1···*Cg*1 distance is 3.927 (2)Å [symmetry code: 1 - *x*,-y, 1 - *z*] and 4.199 (2)Å [symmetry code:-x,-y, 1 - *z*] with a center-to-edge arrangement (Table 2). *Cg*1, *Cg*2 and *Cg*3 are the centroids of N9—C9A—C4A—C5A—C8A, C5A—C5—C6—C7—C8—C8A and C9A—C1—C2—C3—C4—C4A rings, respectively. The carbazole systems are inclined at an angle of about 13.4° to [100] plan.

In conclusion, the crystal structure of an interesting carbazole intermediate has been elucidated. A strong displacement of the N-protecting group out of the plane has been observed. Nevertheless, presence of the *tert*-Butyloxycarbonyl group does not prevent parallel arrangement of carbazole systems by π stacking. Thus, flat similar compounds could be used as anticancer agents through their intercalation effect like ellipticine.

### Experimental

6-Bromo-9-*tert*-butoxycarbonyl-1,4-dimethyl-9*H*-carbazole was prepared by reaction of 6-bromo-1,4-dimethyl-9*H*-carbazole (5.0 g, 18.2 mmol) with di-*tert*-butyl dicarbonate (8.0 g, 36.5 mmol) in the presence of DMAP (4.46 g, 36.5 mmol) and triethylamine (5.1 ml, 36.5 mmol) in acetonitrile (70 ml). The mixture was stirred for 1 h at 0°C, then left at room temperature for 3 h. The residue obtained after removal of the solvent was diluted with EtOAc (100 ml) and shaken with water (2 *x* 100 ml). The residue obtained after an usual work-up was purified by silica gel column chromatography using cyclohexane/ether (7:3) as eluent to give the compound as a yellow solid (63% yield). Transparent crystals suitable for X-ray analysis were grown from an acetonitrile solution at room temperature.

### Refinement

All non-hydrogen atoms were refined anisotropically. The H atoms were refined with fixed geometry, riding on their carrier atoms with *U*_iso_(H) values set at 1.2 (1.5 for methyl H atoms) times *U*_eq_ of the parent atom (C—H = 0.93–0.96 Å) for (I).

### Crystal data

**Table:**

C_19_H_20_BrNO_2_	*Z* = 2
*M**_r_* = 374.27	*F*(000) = 384
Triclinic, *P*1	*D*_x_ = 1.435 Mg m^−^^3^
Hall symbol: -P 1	Mo *K*α radiation, λ = 0.71073 Å
*a* = 7.521 (4) Å	Cell parameters from 9940 reflections
*b* = 9.715 (5) Å	θ = 5.4–57.6°
*c* = 11.930 (6) Å	µ = 2.38 mm^−^^1^
α = 91.10 (4)°	*T* = 291 K
β = 96.40 (4)°	Block, colorless
γ = 90.96 (4)°	0.46 × 0.37 × 0.34 mm
*V* = 865.9 (8) Å^3^	

### Data collection

**Table:**

Bruker–Nonius APEXII Kappa CCD diffractometer	5718 independent reflections
Radiation source: fine-focus sealed tube	4268 reflections with *I* > 2σ(*I*)
graphite	*R*_int_ = 0.025
φ and ω scans	θ_max_ = 31.5°, θ_min_ = 2.1°
Absorption correction: numerical (*SAINT*; Bruker, 2007)	*h* = −11→11
*T*_min_ = 0.378, *T*_max_ = 0.429	*k* = −14→14
37091 measured reflections	*l* = −17→17

### Refinement

**Table:**

Refinement on *F*^2^	Primary atom site location: structure-invariant direct methods
Least-squares matrix: full	Secondary atom site location: difference Fourier map
*R*[*F*^2^ > 2σ(*F*^2^)] = 0.034	Hydrogen site location: inferred from neighbouring sites
*wR*(*F*^2^) = 0.092	H-atom parameters constrained
*S* = 1.02	*w* = 1/[σ^2^(*F*_o_^2^) + (0.0409*P*)^2^ + 0.2555*P*] where *P* = (*F*_o_^2^ + 2*F*_c_^2^)/3
5718 reflections	(Δ/σ)_max_ = 0.001
213 parameters	Δρ_max_ = 0.63 e Å^−^^3^
0 restraints	Δρ_min_ = −0.52 e Å^−^^3^

### Special details

**Table:**

Geometry. All esds (except the esd in the dihedral angle between two l.s. planes) are estimated using the full covariance matrix. The cell esds are taken into account individually in the estimation of esds in distances, angles and torsion angles; correlations between esds in cell parameters are only used when they are defined by crystal symmetry. An approximate (isotropic) treatment of cell esds is used for estimating esds involving l.s. planes.
Refinement. Refinement of *F*^2^ against ALL reflections. The weighted *R*-factor wR and goodness of fit *S* are based on *F*^2^, conventional *R*-factors *R* are based on *F*, with *F* set to zero for negative *F*^2^. The threshold expression of *F*^2^ > σ(*F*^2^) is used only for calculating *R*-factors(gt) etc. and is not relevant to the choice of reflections for refinement. *R*-factors based on *F*^2^ are statistically about twice as large as those based on *F*, and *R*- factors based on ALL data will be even larger.

### Fractional atomic coordinates and isotropic or equivalent isotropic displacement parameters (Å^2^)

**Table:**

	*x*	*y*	*z*	*U*_iso_*/*U*_eq_	
C1	0.3387 (2)	−0.20146 (16)	0.34933 (15)	0.0458 (3)	
C2	0.3632 (2)	−0.30773 (17)	0.42578 (17)	0.0542 (4)	
H2	0.3978	−0.3929	0.3996	0.065*	
C3	0.3389 (2)	−0.29340 (17)	0.53845 (17)	0.0543 (4)	
H3	0.3563	−0.3690	0.5847	0.065*	
C4	0.2894 (2)	−0.16985 (16)	0.58456 (14)	0.0444 (3)	
C4A	0.26189 (18)	−0.06062 (15)	0.51011 (13)	0.0376 (3)	
C5	0.1845 (2)	0.15877 (16)	0.62353 (12)	0.0411 (3)	
H5	0.1796	0.1170	0.6927	0.049*	
C5A	0.22209 (18)	0.08324 (14)	0.52870 (12)	0.0360 (3)	
C6	0.1549 (2)	0.29736 (16)	0.61113 (13)	0.0437 (3)	
C7	0.1590 (2)	0.36264 (16)	0.50904 (14)	0.0466 (3)	
H7	0.1388	0.4567	0.5046	0.056*	
C8	0.1928 (2)	0.28895 (16)	0.41425 (14)	0.0455 (3)	
H8	0.1944	0.3315	0.3452	0.055*	
C8A	0.22440 (19)	0.14932 (15)	0.42488 (12)	0.0377 (3)	
C9	0.2702 (3)	−0.1553 (2)	0.70822 (16)	0.0585 (4)	
H9A	0.3533	−0.0863	0.7419	0.088*	
H9B	0.2945	−0.2417	0.7440	0.088*	
H9C	0.1504	−0.1286	0.7178	0.088*	
C9A	0.28247 (18)	−0.07791 (15)	0.39496 (13)	0.0383 (3)	
C10	0.3831 (3)	−0.2220 (2)	0.23079 (17)	0.0601 (4)	
H10A	0.4583	−0.3004	0.2270	0.090*	
H10B	0.4447	−0.1415	0.2083	0.090*	
H10C	0.2747	−0.2370	0.1812	0.090*	
C11	0.2066 (2)	0.06842 (17)	0.22633 (13)	0.0455 (3)	
C12	0.2220 (3)	0.2419 (2)	0.08037 (15)	0.0595 (4)	
C13	0.3005 (4)	0.1472 (3)	−0.00242 (19)	0.0880 (8)	
H13A	0.2410	0.0588	−0.0042	0.132*	
H13B	0.4259	0.1367	0.0206	0.132*	
H13C	0.2845	0.1858	−0.0762	0.132*	
C14	0.0229 (3)	0.2543 (3)	0.05422 (19)	0.0749 (6)	
H14A	−0.0326	0.1646	0.0537	0.112*	
H14B	−0.0037	0.2946	−0.0184	0.112*	
H14C	−0.0221	0.3116	0.1107	0.112*	
C15	0.3132 (5)	0.3824 (3)	0.0885 (2)	0.0956 (9)	
H15A	0.2879	0.4284	0.0181	0.143*	
H15B	0.4401	0.3719	0.1047	0.143*	
H15C	0.2695	0.4360	0.1477	0.143*	
Br1	0.10889 (3)	0.40593 (2)	0.738576 (16)	0.06549 (9)	
O1	0.12304 (19)	−0.01560 (14)	0.16710 (11)	0.0611 (3)	
O2	0.26615 (18)	0.19081 (13)	0.19661 (10)	0.0537 (3)	
N9	0.25825 (17)	0.05083 (13)	0.34212 (10)	0.0408 (3)	

### Atomic displacement parameters (Å^2^)

**Table:**

	*U*^11^	*U*^22^	*U*^33^	*U*^12^	*U*^13^	*U*^23^
C1	0.0374 (7)	0.0426 (7)	0.0575 (9)	0.0008 (6)	0.0074 (6)	−0.0080 (7)
C2	0.0496 (9)	0.0381 (8)	0.0750 (12)	0.0043 (7)	0.0072 (8)	−0.0050 (7)
C3	0.0532 (9)	0.0395 (8)	0.0700 (11)	0.0029 (7)	0.0048 (8)	0.0088 (7)
C4	0.0380 (7)	0.0428 (7)	0.0521 (8)	−0.0002 (6)	0.0034 (6)	0.0072 (6)
C4A	0.0305 (6)	0.0376 (7)	0.0448 (7)	0.0003 (5)	0.0040 (5)	0.0011 (5)
C5	0.0416 (7)	0.0454 (8)	0.0365 (7)	0.0035 (6)	0.0048 (6)	0.0014 (6)
C5A	0.0312 (6)	0.0383 (7)	0.0386 (7)	0.0023 (5)	0.0030 (5)	0.0015 (5)
C6	0.0435 (8)	0.0458 (8)	0.0418 (7)	0.0071 (6)	0.0044 (6)	−0.0055 (6)
C7	0.0504 (9)	0.0386 (7)	0.0507 (8)	0.0091 (6)	0.0041 (7)	−0.0004 (6)
C8	0.0534 (9)	0.0422 (7)	0.0410 (7)	0.0075 (6)	0.0042 (6)	0.0049 (6)
C8A	0.0359 (7)	0.0395 (7)	0.0376 (6)	0.0027 (5)	0.0032 (5)	−0.0010 (5)
C9	0.0647 (11)	0.0576 (10)	0.0540 (10)	0.0060 (8)	0.0070 (8)	0.0172 (8)
C9A	0.0324 (6)	0.0377 (7)	0.0446 (7)	−0.0002 (5)	0.0044 (5)	−0.0006 (5)
C10	0.0612 (11)	0.0584 (10)	0.0620 (11)	0.0077 (8)	0.0145 (8)	−0.0156 (8)
C11	0.0471 (8)	0.0499 (8)	0.0400 (7)	0.0040 (7)	0.0068 (6)	−0.0013 (6)
C12	0.0760 (12)	0.0653 (11)	0.0388 (8)	0.0052 (9)	0.0114 (8)	0.0088 (7)
C13	0.110 (2)	0.109 (2)	0.0513 (11)	0.0309 (16)	0.0346 (12)	0.0106 (12)
C14	0.0836 (15)	0.0850 (15)	0.0567 (11)	0.0204 (12)	0.0062 (10)	0.0129 (11)
C15	0.129 (2)	0.0829 (17)	0.0745 (16)	−0.0223 (16)	0.0062 (15)	0.0314 (13)
Br1	0.08728 (16)	0.05996 (12)	0.05042 (11)	0.01706 (10)	0.01285 (9)	−0.01170 (8)
O1	0.0717 (8)	0.0602 (8)	0.0484 (7)	−0.0039 (6)	−0.0046 (6)	−0.0062 (6)
O2	0.0680 (8)	0.0550 (7)	0.0382 (5)	−0.0045 (6)	0.0059 (5)	0.0051 (5)
N9	0.0451 (7)	0.0402 (6)	0.0374 (6)	0.0032 (5)	0.0056 (5)	−0.0012 (5)

### Geometric parameters (Å, °)

**Table:**

C1—C2	1.392 (3)	C9—H9C	0.9600
C1—C9A	1.399 (2)	C9A—N9	1.417 (2)
C1—C10	1.499 (3)	C10—H10A	0.9600
C2—C3	1.381 (3)	C10—H10B	0.9600
C2—H2	0.9300	C10—H10C	0.9600
C3—H3	0.9300	C11—O1	1.193 (2)
C4—C3	1.384 (3)	C11—O2	1.332 (2)
C4—C4A	1.400 (2)	C11—N9	1.407 (2)
C4—C9	1.502 (3)	C12—O2	1.486 (2)
C4A—C9A	1.407 (2)	C12—C13	1.511 (3)
C5—C5A	1.394 (2)	C12—C15	1.514 (3)
C5—H5	0.9300	C13—H13A	0.9600
C5A—C8A	1.408 (2)	C13—H13B	0.9600
C5A—C4A	1.452 (2)	C13—H13C	0.9600
C6—C5	1.376 (2)	C14—C12	1.502 (3)
C6—C7	1.387 (2)	C14—H14A	0.9600
C7—C8	1.377 (2)	C14—H14B	0.9600
C7—H7	0.9300	C14—H14C	0.9600
C8—C8A	1.387 (2)	C15—H15A	0.9600
C8—H8	0.9300	C15—H15B	0.9600
C8A—N9	1.408 (2)	C15—H15C	0.9600
C9—H9A	0.9600	Br1—C6	1.9004 (18)
C9—H9B	0.9600		
			
C1—C2—H2	118.3	C8A—N9—C9A	108.07 (12)
C1—C9A—C4A	122.57 (15)	C9A—C1—C10	125.22 (16)
C1—C9A—N9	128.35 (14)	C9A—C4A—C5A	107.15 (13)
C1—C10—H10A	109.5	H9A—C9—H9B	109.5
C1—C10—H10B	109.5	H9A—C9—H9C	109.5
C1—C10—H10C	109.5	H9B—C9—H9C	109.5
C2—C1—C9A	114.58 (16)	H10A—C10—H10B	109.5
C2—C1—C10	120.08 (16)	H10A—C10—H10C	109.5
C2—C3—C4	122.00 (17)	H10B—C10—H10C	109.5
C2—C3—H3	119.0	C11—O2—C12	121.26 (14)
C3—C2—C1	123.47 (16)	C11—N9—C8A	122.65 (13)
C3—C2—H2	118.3	C11—N9—C9A	125.02 (13)
C3—C4—C4A	116.27 (16)	C12—C13—H13A	109.5
C3—C4—C9	121.07 (16)	C12—C13—H13B	109.5
C4—C3—H3	119.0	C12—C13—H13C	109.5
C4—C4A—C9A	121.03 (14)	C12—C14—H14A	109.5
C4—C4A—C5A	131.72 (15)	C12—C14—H14B	109.5
C4—C9—H9A	109.5	C12—C14—H14C	109.5
C4—C9—H9B	109.5	C12—C15—H15A	109.5
C4—C9—H9C	109.5	C12—C15—H15B	109.5
C4A—C4—C9	122.65 (16)	C12—C15—H15C	109.5
C4A—C9A—N9	108.67 (13)	C13—C12—C15	111.9 (2)
C5—C5A—C8A	119.61 (14)	H13A—C13—H13C	109.5
C5—C5A—C4A	133.06 (14)	H13B—C13—H13C	109.5
C5—C6—C7	122.74 (14)	H13A—C13—H13B	109.5
C5—C6—Br1	119.29 (12)	C14—C12—C13	112.2 (2)
C5A—C5—H5	121.2	C14—C12—C15	110.9 (2)
C5A—C8A—N9	108.75 (13)	H14A—C14—H14B	109.5
C6—C5—C5A	117.59 (14)	H14A—C14—H14C	109.5
C6—C5—H5	121.2	H14B—C14—H14C	109.5
C6—C7—H7	119.8	H15A—C15—H15C	109.5
C7—C6—Br1	117.97 (12)	H15A—C15—H15B	109.5
C7—C8—C8A	117.97 (15)	H15B—C15—H15C	109.5
C7—C8—H8	121.0	O1—C11—O2	127.27 (16)
C8—C7—C6	120.33 (15)	O1—C11—N9	123.67 (16)
C8—C7—H7	119.8	O2—C11—N9	109.06 (14)
C8—C8A—C5A	121.75 (14)	O2—C12—C14	110.07 (16)
C8—C8A—N9	129.48 (14)	O2—C12—C13	109.40 (17)
C8A—C5A—C4A	107.33 (13)	O2—C12—C15	101.86 (17)
C8A—C8—H8	121.0		
			
C1—C9A—N9—C11	30.8 (2)	C8A—N9—C11—O1	−128.47 (18)
C8—C8A—N9—C11	−22.4 (2)	C9A—C4A—C4—C9	179.52 (15)
C9A—N9—C11—O2	−154.38 (14)	C2—C3—C4—C9	−177.79 (17)
C9A—N9—C11—O1	25.5 (3)	C3—C2—C1—C10	174.71 (17)
C8A—N9—C11—O2	51.6 (2)	C4A—C9A—C1—C10	−172.72 (15)

### Hydrogen-bond geometry (Å, °)

**Table:**

*D*—H···*A*	*D*—H	H···*A*	*D*···*A*	*D*—H···*A*
C8—H8···O2	0.93	2.33	2.863 (3)	116

### Table 2 π–π interactions (Å, °).

*Cg*1, *Cg*2 and *Cg*3 are the centroids of the N9–C9A–C4A–C5A–C8A, C9A–C1–C2–C3–C4–C4A and C5A–C5–C6–C7–C8–C8A rings, respectively, ccd is the distance between ring centroids, sa is the mean slippage angle (angle subtended by the intercentroid vector to the plane normal) and ipd is the mean interplanar distance (distance from one plane to the neighbouring centroid). For details, see Janiak (2000).

**Table:**

Group 1/group 2	ccd	sa	ipd
*Cg*2/*Cg*3^i^	3.755 (2)	24	3.532 (1)
*Cg*3/*Cg*2^i^	3.755 (2)	20	3.433 (1)
*Cg*1/*Cg*1^i^	3.927 (2)	22	3.638 (1)
*Cg*2/*Cg*3^ii^	3.811 (2)	18	3.654 (1)
*Cg*3/*Cg*2^ii^	3.811 (2)	16	3.626 (1)
*Cg*1/*Cg*1^ii^	4.199 (2)	32	3.578 (1)

Symmetry codes: (i) -x+1, -y, -z+1; (ii) -x, -y, -z+1.
